# A User-Adaptive Algorithm for Activity Recognition Based on K-Means Clustering, Local Outlier Factor, and Multivariate Gaussian Distribution

**DOI:** 10.3390/s18061850

**Published:** 2018-06-06

**Authors:** Shizhen Zhao, Wenfeng Li, Jingjing Cao

**Affiliations:** School of Logistics Engineering, Wuhan University of Technology, Wuhan 430070, China; henrylzqlj@whut.edu.cn (S.Z.); bettycao@whut.edu.cn (J.C.)

**Keywords:** human activity recognition, user-adaptive algorithm, K-Means clustering, local outlier factor, multivariate Gaussian distribution, personalized classifier

## Abstract

Mobile activity recognition is significant to the development of human-centric pervasive applications including elderly care, personalized recommendations, etc. Nevertheless, the distribution of inertial sensor data can be influenced to a great extent by varying users. This means that the performance of an activity recognition classifier trained by one user’s dataset will degenerate when transferred to others. In this study, we focus on building a personalized classifier to detect four categories of human activities: light intensity activity, moderate intensity activity, vigorous intensity activity, and fall. In order to solve the problem caused by different distributions of inertial sensor signals, a user-adaptive algorithm based on K-Means clustering, local outlier factor (LOF), and multivariate Gaussian distribution (MGD) is proposed. To automatically cluster and annotate a specific user’s activity data, an improved K-Means algorithm with a novel initialization method is designed. By quantifying the samples’ informative degree in a labeled individual dataset, the most profitable samples can be selected for activity recognition model adaption. Through experiments, we conclude that our proposed models can adapt to new users with good recognition performance.

## 1. Introduction 

Human activity recognition (HAR) is a research field that plays an important role in healthcare systems [1]. It can help people to build a healthy lifestyle with regular physical activities. At present, the methods for HAR are mainly based on computer vision [2] and wearable sensors [3]. The methods based on computer vision use cameras to monitor the movements of a human body and classify the types of human body activities by means of specific algorithms [4,5]. With cameras functioning as the human visual system, these methods use a specific algorithm to simulate the process of human brain judgment. The advantage of camera assistance is that multiple subjects can be detected at the same time without the aid of other devices [6]. Although the accuracy rate is high in some cases, the practical value of this method is undermined by its high requirements of equipment and its tendency to suffer high interference from environmental factors [7]. Moreover, camera-based methods can generate privacy issues [8]. As for the methods based on wearable sensors, with the development of micro-electro-mechanical system (MEMS) technology, human body motion sensors have been miniaturized, thus becoming more flexible and more affordable [9]. Inertial sensor-based HAR can be mainly divided into two steps: feature extraction and classification [9].

Classification techniques commonly used for activity identification based on sensory data were reviewed in [10,11]. Most previous activity recognition research studies created classification models offline from the training dataset, which contained the data of various people, and transferred these models to different users to validate classification performance. This limits the performance of human activity recognition because the distribution of sensory data is influenced greatly by varying users [12,13]. This is because walking for one user may well be running for another in some cases; therefore, HAR personalization is important for constructing a robust HAR system. In fact, it is hard to generate a user-specific model because of the scarcity of labeled data [14]. Some studies utilized a data generator [15] for data augmentation or designed a framework (e.g., personal digital assistant) [16] for genuine data collection. In contrast, we attempt to design an automatic annotation method for constructing a personalized labeled dataset. 

In this paper, we aim to build an adaptive algorithm based on K-Means clustering, local outlier factor (LOF), and multivariate Gaussian distribution (MGD) for activity recognition. We focus on the personalized classifier about its ability to recognize four human activities: light intensity activity (LIA), moderate intensity activity (MIA), vigorous intensity activity (VIA), and fall. The developed algorithm deals with user-specific sensory data to learn and recognize personalized users' activities. Manual data labeling for each user is usually impractical, inconvenient, and time-consuming. To automatically annotate a specific user’s dataset, a novel initialization method of a K-Means algorithm is presented. The core idea of this method is to set initial centroids of unclustered dataset as K centroids (with labels) of pre-collected activity dataset. Then, a method based on LOF is proposed to select high confidence samples for personalizing three MGD models. The three MGD models can estimate the probabilities of real-time human activity states including LIA, MIA, and VIA. To further recognize falls, thresholds are set to distinguish a fall from VIA. 

The rest of this paper is organized as follows: in Section 2, we present some relative studies that work on physical activity intensity recognition, fall detection, and user-adaptive recognition models without manual interruption. In Section 3, we describe our adaptive algorithm for activity recognition. In Section 4, we set up the experiments and analyze the results. We conclude this paper in Section 5. 

## 2. Related Works

In this section, an overview of three main topics related to this paper is given: (1) physical activity intensity recognition; (2) fall detection; and (3) user-adaptive recognition models without manual interruption.

### 2.1. Physical Activity Intensity Recognition

Liu et al. [17] proposed the Accumulated Activity Effective Index Reminder (AAEIReminder), a fuzzy logic prompting mechanism, to help users to manage physical activity. AAEIReminder can detect activity levels using a C4.5 decision tree algorithm and infer activity situations (e.g., the amount of physical activity, days spent exercising) through fuzzy logic. Then it decides whether the situation requires a prompt and how much exercise should be prompted. Liu et al. [18] assessed physical activity and the corresponding energy expenditure through multisensory data fusion (i.e., acceleration and ventilation) based on SVMs. Their experimental results showed that the proposed method is more effective in physical activity assessment and detecting energy expenditure compared with single accelerometer-based methods. Jung et al. [19] used fuzzy logic and an SVM algorithm to enable the classification of datasets from bio-sensors. Then, a decision tree and random forest algorithm was utilized to identify the mental stress level. Finally, a novel activity assessment model based on an expectation maximization (EM) algorithm was proposed to assess users’ mental wellness so as to recommend them proper activity. Fahim et al. [20] classified acceleration data from smartphones, using the non-parametric nearest neighbor algorithm, to analyze sedentary lifestyles. The classification process was conducted on the cloud platform, which facilitates users to monitor their long-term sedentary behavior. Ma et al. [21] designed a novel activity level assessment approach for people who suffer from sedentary lifestyles. With their method, users’ postures and body swings can be detected using a J48 decision tree algorithm and sensory data from a smart cushion. Then a method based on the estimation of activity assessment index (AAI) is used to further recognize the activity levels. The aforementioned methods utilize a generic classifier without personalization to assess physical activity. Thus, their performance is limited because the distribution sensory data vary among different people, which leads to inevitable cross-person error. 

### 2.2. Fall Detection

Tong et al. [22] proposed a method based on the hidden Markov model (HMM), using tri-axial accelerations, to detect falls. They used the acceleration time series of fall processes before the collision to train the HMM model. Thus, their trained model can not only detect falls but also evaluate fall risks. Bourke et al. [23] described a threshold-based algorithm for fall detection, using the bi-axial angular velocity. Three thresholds for resultant angular velocity, resultant angular acceleration, and resultant angle change were set to distinguish falls from activities of daily living. Sucerquia et al. [24] presented a novel fall detection approach based on a Kalman filter and a non-linear classification feature, using data from a tri-axial accelerometer. Their methodology required a low sampling frequency of only 25 Hz. The experimental results showed that their proposed method has low computational complexity and is robust among embedded systems. Khojasteh et al. [25] compared the performance of threshold-based algorithms and various machine learning algorithms in detecting falls, using data from waist-located tri-axial accelerometers. The experimental results showed that the machine learning algorithms outperformed the threshold-based algorithms. Moreover, among the selected machine learning algorithms, support vector machines provide the highest combination of sensitivity and specificity. Mao et al. [26] re-identified an acceleration threshold of 2.3 g and verified the best sensor location (i.e., waist) of the human body for fall detection. Shi et al. [27] uses J48 decision tree, which is an efficient algorithm derived from C4.5 decision tree [28], to detect falls. The aforementioned methods [22,23,24,25,26,27] do not consider the effect of personalization on fall detection. Medrano et al. [8] evaluated four algorithms (nearest neighbor (NN), local outlier factor (LOC), one-class support vector machine (One-Class SVM), and SVM) after personalization used as fall detectors to boost their performance when compared to their non-personalized versions. The experimental results showed that there is a general trend towards an increase in performance by detector personalization, but the effect depends on the individual being considered. However, manual labeling is needed in its personalization process, which is impractical in real applications. 

### 2.3. User-Adaptive Recognition Model without Manual Interruption

For HAR personalization, manual data labeling for each user is usually impractical, inconvenient, and time-consuming. Some studies automatically personalized their HAR model without human intervention. Viet et al. [29] combined an SVM classifier with K-medoids clustering to build a personalized activity recognition model. Moreover, each user can update the model using his new activities independently. However, training an SVM model requires intensive computation which may not be allowed in a lightweight embedded device. Zhao et al. [30] presented a user-adaptive HAR model adaptation by combining a K-Means clustering algorithm with a decision tree. The outputs of the trained decision tree are organized by the K-Means algorithm. Then, the decision tree is re-trained by the dataset for HAR personalization. Deng et al. [31] presented a fast and accurate cross-person HAR algorithm, which is known as Transfer learning Reduced Kernel Extreme Learning Machine (TransRKELM). Reduced Kernel Extreme Learning Machine (RKELM) is used to build an initial activity recognition model. Then, Online Sequential-Reduced Kernel Extreme Learning Machine (OS-RKELM) is applied to reconstruct the previous model for personalization. Wen et al. [32] utilized AdaBoost to choose the most profitable features automatically during the adaptation process. The initial model is trained by a pre-collected dataset. Dynamically available data sources are then used to adapt and refine the model. Fallahzadeh et al. [33] proposed a cross-person algorithm called Knowledge Fusion-Based Cross-Subject Transfer Learning. First, a source dataset is used to construct an initial activity recognition model (i.e., source model). Second, the source model is tested on a target dataset, which is collected from a new user, to assign supervised label predictions. Third, the highly similar samples are annotated as a particular activity category if their degree of correlation is higher than a threshold set by a greedy algorithm. Then, the samples of the target dataset are annotated depending on the information acquired from both source and target views to build up an individual dataset. Lastly, an activity recognition model is trained by the personalized labeled dataset. Siirtola et al. [34] used the Learn++ algorithm, which can utilize any classifier as a base classifier, to personalize human activity recognition models. They compared three different base classifiers: quadratic discriminant analysis (QDA), classification and regression tree (CART), and linear discriminant analysis (LDA). The experiment results showed that even a small personalized dataset can improve the classification accuracy, with QDA by 2.0%, CART by 2.3%, and LDA by 4.6%.

Our method is different from those of the abovementioned studies in numerous aspects. Instead of utilizing a generic model to classify or annotate new users’ sensory data, we propose a method based on a clustering algorithm to cluster and annotate instances from a new user automatically. Existing HAR adaption methods rely on a trained model that can be updated and adapted to new users, while we consider training a model completely based on new users’ sensory data without others’ information. Previous activity recognition models usually select high confidence samples for personalization, while we propose a method based on relative density to remove low confidence samples in each data cluster.

## 3. User-Adaptive Algorithm for Activity Recognition

**Figure 1:**
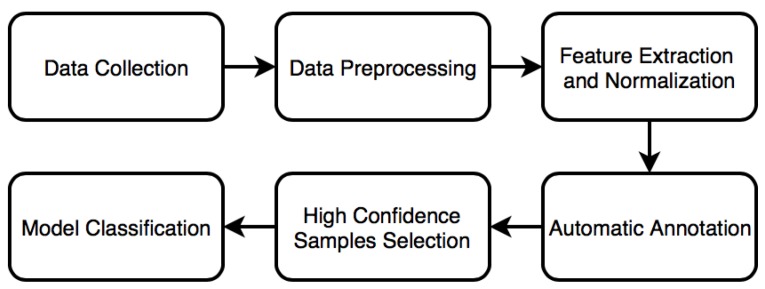
The process of establishing our proposed user-adaptive algorithm.

The aim of this research is to design a user-adaptive algorithm for recognizing four categories of human activities: LIA, MIA, VIA, and fall. The process of establishing our proposed method, as shown in Figure 1, includes data collection, data preprocessing, feature extraction and normalization, automatic annotation (K-Means clustering), high confidence samples selection, and model classification. Each step is detailed in the following subsections.

### 3.1. Data Preprocessing and Feature Extraction

Acceleration and angular velocity signals [35,36,37] of the human body can describe states of human activity. Acceleration signals are separated by a Butterworth low-pass filter into gravity and body acceleration. The filter with a 0.3-Hz cutoff frequency is utilized, because the gravitational force has only low frequency components [31]. The data flow is segmented into small windows to deal with large amounts of data, thus facilitating the study and analysis. The size of the sliding window T and the sampling frequency f in our study are set to 1 second and 50 Hz, respectively. Therefore, the *k*th data units are: $X_{k}=\left\{x_{n},x_{n+1},x_{n+2},\ldots ,x_{n+48},x_{n+49}\right\},\;1\leq k\leq M$, where *M* is the total number of data windows. Through extensive experiments, a fall process was established to take about 300 ms [38], which starts from the beginning of losing balance and continues to the collision of body with lower objects. In order not to cut off the data of the fall process, each data window overlaps with the previous window by 50%. Thus, we set n=25×(k−1). Each sampling point of collected raw data is: $\;x_{r}=\left(a_{rx},a_{ry},a_{rz},\omega_{rx},\omega_{ry},\omega_{rz}\right)$. According to the Equation (1), we preprocess the raw data and obtain every sample point: $x=\left(a_{x},a_{y},a_{z},\omega_{x},\omega_{y},\omega_{z}\right)$:

(1) $$\{\begin{array}{l} a_{x}=a_{rx}/k_{ax}+b_{ax}\\ a_{y}=a_{ry}/k_{ay}+b_{ay}\\ a_{z}=a_{rz}/k_{az}+b_{az}\\ \omega_{x}=\omega_{rx}/k_{\omega x}+b_{\omega x}\\ \omega_{y}=\omega_{ry}/k_{\omega y}+b_{\omega y}\\ \omega_{z}=\omega_{rz}/k_{\omega z}+b_{\omega z} \end{array}$$

$k_{ax},\;k_{ay},\;k_{az},\;k_{\omega x},\;k_{\omega y},\;k_{\omega z}$ are the sensitivity coefficients of three axes. Sensitivity coefficients measure the degree of change of an accelerometer or gyroscope in response to unit acceleration or unit angular velocity changes. $b_{ax},\;b_{ay},\;b_{az},\;b_{\omega x},\;b_{\omega y},\;b_{\omega z}$ are their zero drift values. Because the zero drift values are small, their impact is negligible and they were thus ignored in this study. 

The quality of feature extraction determines the upper limit of classification performance, and good features can facilitate the process of subsequent classification. Generally, the methods of extracting features from an inertial sensor signal fall into the following three categories: time domain analysis, frequency domain analysis, and time-frequency analysis. Among them, the time domain features are the most commonly used, followed by the frequency domain features. In this study, only the time domain features are extracted because of their lower computational complexity compared with that of the frequency domain feature and the time-frequency feature. The magnitude of synthesized acceleration and angular velocity can be expressed as: $a=\sqrt{a_{x}{}^{2}+a_{y}{}^{2}+a_{z}{}^{2}}$ and $\omega =\sqrt{\omega_{x}{}^{2}+\omega_{y}+\omega_{z}{}^{2}}$. From each window, a vector of 13 features is obtained by calculating variables in the time domain. The mean, standard deviation, energy, mean-crossing rate, maximum value, and minimum value are extracted from the magnitude of synthesized acceleration and angular velocity. In addition, one extra feature, termed tilt angle (*TA*), can be extracted using Equation (2):

(2) $$TA=\sqrt{{\left(\int \omega_{x}dt\right)}^{2}+{\left(\int \omega_{z}dt\right)}^{2}}$$

### 3.2. Automatic Annotation Using K-Means Algorithm

In this study, a K-Means algorithm [39] is used to cluster and annotate users’ data. The K-Means algorithm has many advantages such as small computational complexity, high efficiency for large datasets, and high linearity of time complexity. However, its clustering results and iteration times are up to the initial cluster centers and the algorithm can function very slowly to converge with a bad initialization [30]. In our solution, a dataset with great similarity to the unclustered dataset is pre-collected. Then the initial center points (with labels) are set to be the cluster centers of this dataset before conducting K-Means clustering steps on the unclustered dataset. After clustering step iterations, the clusters are annotated automatically because of the labeled initial points. The limitation of this method concerns whether a similar dataset can be collected. In this study, the similar dataset can be obtained by experiments.

Let the unclustered dataset $X=\{x_{i}|i=1,\ldots ,M\}$ be a dataset having K clusters, let $C=\{c_{k}|k=1,\ldots ,K\}$ be a set of K cluster centers, and let $S_{k}=\{x_{j}^{S_{k}}|j=1,\ldots ,m^{S_{k}}\}$ be a set of samples that belong to the kth cluster. In order to find the initial centroids that are close to the optimal result, the data of the K clusters are collected previously through experiments. Let $X^{p}=\{x_{i}^{p}|i=1,\ldots ,N\}$ be a pre-collected dataset, let $C^{p}=\{c_{k}^{p}|k=1,\ldots ,K\}$ be a set of K cluster centers of this dataset, and let $S_{k}^{p}=\{x_{j}^{S_{k}^{p}}|j=1,\ldots ,n^{S_{k}}\}$ be a set of samples that belong to the kth cluster. The steps of the K-Means clustering we propose are summarized as follows:

(1) Initialization step: Calculate the $c_{k}$ centers as:
  (3) $$c_{k}^{0}=c_{k}^{p}=\frac{\sum x_{j}^{S_{k}^{p}}}{\left|S_{k}^{p}\right|}$$
  where $\left|S_{k}^{p}\right|$ is the number of samples in the *k*th cluster of a pre-collected dataset.
(2) Assignment step: Determine the category of the patterns in one of the K clusters whose mean has the least squared Euclidean distance.
  (4) $$S_{k}^{\left(t\right)}=\left\{x_{i}:∥x_{i}-c_{k}^{\left(t\right)}{∥}^{2}\leq ∥x_{i}-c_{p}^{\left(t\right)}∥\forall j,1\leq p\leq K\right\}$$
  where each $x_{q}$ is assigned to exactly one $S_{k}^{\left(t\right)}$.
(3) Update step: Calculate the new $c_{k}^{\left(t+1\right)}$ as:
  (5) $$c_{k}^{\left(t+1\right)}=\frac{\sum x_{j}^{S_{k}}}{\left|S_{k}^{\left(t\right)}\right|}$$
  $\left|S_{k}^{\left(t\right)}\right|$ is the number of samples in the *k*th cluster of the unclustered dataset.
(4) Repeat steps 2 and 3 until there is no change in the assignment step.

The main difference between this K-Means algorithm and the traditional one is that the initial centers we used are labeled and, due to the similarity of different people’s activity data, they are close to the expected optimal results.

### 3.3. High Confidence Samples Selection

After conducting automatic annotation on a new user’s dataset, confident samples should be selected for MGD model training. Most previous studies [29,30,31] only selected a certain number of samples in each cluster or chose the samples with high classification confidence. However, these methods ignored the effect of relative density on high confidence samples selection. In our study, some outliers with low relative density are removed from each cluster. Researchers have designed algorithms that take the relative density of a given data instance into consideration to compute the outlier score. LOF [40], a top-notch technique, allows the score to be equal to the average local density ratio of the K-nearest neighbors of the instance and the local density ratio of the data instance itself. Specifically, the LOF score depends on the values of reachability distance and reachability density. In our study, each sample of a new user has its label after automatic annotation. Also, LOF is used to remove the outliers of each cluster. Accordingly, the K-nearest neighbors of a sample only include samples in its belonging cluster. Because LOF utilizes the labels of the dataset in our study, we term it label-based local outlier factor (LLOF). If $d_{kNNbc}\left(C\right)$ is the distance to the K-nearest neighbor in its belonging cluster, then the reachability distance between two samples is defined as follows:

(6) $$d_{reach}\left(C,D\right)=\max\left(\;d_{kNNbc}\left(C\right),\;d\left(C,D\right)\right)$$

where d(C,D) represents the Euclidean distance between samples *C* and *D*.

Taking the maximum in the definition of the reachability distance reduces the statistical fluctuations of d(C,D) for records that are close to *C*. The reachability density is defined as follows:

(7) $$\rho_{reach}\left(C\right)=\frac{\left|N_{kbc}\left(C\right)\right|}{\sum_{D\in N_{kbc}\left(C\right)}d_{reach}}$$

where $N_{kbc}\left(C\right)$ is the K-neighborhood of record *C*, meaning the set of its K-nearest neighbors in its belonging cluster, and $\left|N_{k}\left(C\right)\right|$ is its number. Then, the LLOF score is defined as follows:

(8) $$LLOF\left(C\right)=\frac{\sum_{D\in N_{kbc}\left(C\right)}\frac{\rho_{reach}\left(D\right)}{\rho_{reach}\left(C\right)}}{\left|N_{kbc}\left(C\right)\right|}$$

The LOF score is expected to be close to 1 inside a tight cluster, while it increases for outliers [40]. A threshold $\varepsilon_{1}$ is set to filter the outliers.

### 3.4. Training Phase

For the classification technique selection, the rarity of occurrences of falls are an important factor under consideration. Falls occur infrequently and diversely, leading to a lack of related data for training the classifiers [41]. Alternatively, artificial fall data can be collected in controlled laboratory settings, but they may not be the best representatives of actual falls [42]. Moreover, classification models built with artificial falls are more likely to suffer from the problem of over-fitting, caused by time series dataset imbalance [43,44], and may poorly generalize actual falls. In this case, we propose an MGD-based classifier, which does not require fall data in training phase, for detecting LIA, MIA, VIA, and fall. Because it does not require high computational complexity (in the case of low dimensions) and only a few parameters need to be computed in its training phase, it can easily be implemented in wearable embedded systems.

Let $S_{k}=\{x_{j}^{S_{k}}|j=1,\ldots ,m^{k}\}$ be the kth cluster in the training set X and let each sample $x_{j}^{S_{k}}$ be an dimensional feature vector: $x_{j}^{S_{k}}={\left(x_{1}^{S_{k}},x_{2}^{S_{k}},\ldots ,x_{n}^{S_{k}}\right)}^{T}$. The training phase of MGD using $S_{k}$ is summarized as follows:

(9) $$\mu_{k}=\frac{1}{m^{k}}\sum_{j=1}^{m^{k}}\;x_{j}^{S_{k}}$$

(10) $$\Sigma_{k}=\frac{1}{m^{k}}\sum_{J=1}^{m^{k}}\left(x_{j}^{S_{k}}-\mu_{k}\right){\left(x_{j}^{S_{k}}-\mu_{k}\right)}^{T}$$

Given a new example x, p(x) is computed as:

(11) $$p_{k}\left(x\right)=\frac{1}{{\left(2\pi \right)}^{\frac{n}{2}}{\left|\Sigma_{k}\right|}^{\frac{1}{2}}}exp\left(-\frac{1}{2}{\left(x-\mu_{k}\right)}^{T}\Sigma^{-1}\left(x-\mu_{k}\right)\right)$$

The output p(x) can be used to estimate the probability that a new example belongs to the same class of $S_{k}$. The example x is determined to be an anomaly if p(x)<ε (ε is a threshold value).

Assume that we have a pre-collected dataset $X^{p}$ that is similar to an unlabeled dataset X in distribution, and that both datasets include the same data cluster: LIA, MIA, and VIA. The two datasets are collected from different subjects. Since one MGD model only fits one specific class, three MGD models should be trained in order to identify the class of new samples into LIA, MIA, and VIA. When there is a new sample, we assign the class label of maximum of $p_{i}\left(x\right)$.Through extensive experiments, falls are classified into VIA in this stage. Then, thresholds are set to distinguish fall from VIA, which are introduced in Section 3.5. The steps of the training phase of our proposed classifier are summarized as follows (Algorithm 1):
**Algorithm 1** **Training phase****Input:** raw dataset without annotation $Y=\{y_{i}|i=1,\ldots ,M\}$, prelabeled dataset $X^{p}=\left\{x_{i}^{p}|i=1,\ldots ,N\right\},\;C^{p}=\left\{c_{k}^{p}|k=1,\ldots ,K\right\},\;S_{k}^{p}=\{x_{j}^{S_{k}^{p}}|j=1,\ldots ,n^{S_{k}}\}$, max iterations *T*, nearest neighbor number k, outlier threshold $\varepsilon_{1}$**Output:** personalized MGD models $p_{1}\left(x\right),\;p_{2}\left(x\right),\;\mathrm{and}\;p_{3}\left(x\right)$Preprocess the dataset $Y=\{y_{i}|i=1,\ldots ,M\}$ through the Butterworth low-pass filter and dividing by sensitivity coefficient k.Segment the data into overlapping windows.Extract the features and normalization.Save the results from step 1 and step 2 as $X=\{x_{i}|i=1,\ldots ,M\}$.Initialize K-Means clustering centroids $c_{k}^{0}$ by Equation (3).For *t* = 1 to *T* do the following: Assign samples that belong to the kth cluster $S_{k}$ by Equation (4). Update kth centroids $c_{k}^{\left(t+1\right)}$ by Equation (5). If there is no change in the assignment step  break end ifend forFor *i* = 1 to *M* do the following: $score_{i}=LLOF\left(x_{i}\right),\;x_{i}\in X$ if $score_{i}>\varepsilon_{1}$  remove $x_{i}$ from X end ifend forUsing Equation (9) and (10), compute $\mu_{1},\;\Sigma_{1}$ by the LIA cluster $S_{1}=\{x_{J}^{S_{1}}|j=1,\ldots ,m^{S_{1}}\}$, compute $\mu_{2},\;\Sigma_{2}$ by the MIA cluster $S_{2}=\{x_{j}^{S_{2}}|j=1,\ldots ,m^{S_{2}}\}$, and compute $\mu_{3},\;\Sigma_{3}$ by the VIA cluster $S_{3}=\{x_{j}^{S_{3}}|j=1,\ldots ,m^{S_{3}}\}$.Establish the personalized MGD models: $p_{1}\left(x\right)=\frac{1}{{\left(2\pi \right)}^{\frac{n}{2}}{\left|\;\Sigma_{1}\right|}^{\frac{1}{2}}}exp\left(-\frac{1}{2}{\left(x-\mu_{1}\right)}^{T}\;\Sigma_{3}{}^{-1}\left(x-\mu_{1}\right)\right)$, $p_{2}\left(x\right)=\frac{1}{{\left(2\pi \right)}^{\frac{n}{2}}{\left|\Sigma_{2}\right|}^{\frac{1}{2}}}exp\left(-\frac{1}{2}{\left(x-\mu_{2}\right)}^{T}\Sigma_{2}{}^{-1}\left(x-\mu_{2}\right)\right)$, and $p_{3}\left(x\right)=\frac{1}{{\left(2\pi \right)}^{\frac{n}{2}}{\left|\;\Sigma_{3}\right|}^{\frac{1}{2}}}exp\left(-\frac{1}{2}{\left(x-\mu_{3}\right)}^{T}\;\Sigma_{3}{}^{-1}\left(x-\mu_{3}\right)\right)$

### 3.5. Testing Phase

The function of our proposed algorithm is to classify human activities into four categories: LIA, MIA, VIA, and fall. VIA and fall can be attributed to the same category due to its large signal variance in a sampling period. Then, the activity state can be determined using Equation (12) and two thresholds, $\varepsilon_{1}$ (threshold of $p_{3}\left(x\right)$) and $\varepsilon_{2}$ (threshold of *TA*):

(12) $$i^{\prime }=\underset{i}{argmax}p_{i}\left(x\right)$$

The $\varepsilon_{2}$ is employed to reassure the tilt angle change in a fall movement. If i'=1, the real-time activity is determined to be LIA; if i'=2, the real-time activity is determined to be MIA; if $i'=3\;\&\;p_{3}\left(x\right)>\varepsilon_{2}\;\&\;TA<\varepsilon_{3}$, the real-time activity is determined to be VIA; if $i'=3\;\&\;p_{3}\left(x\right)\leq \varepsilon_{2}\;\&\;TA\geq \varepsilon_{3}$, the real-time activity is determined to be fall. The overall algorithm is summarized as follows (Algorithm 2):
**Algorithm 2** **Classifier****Input:** real-time data A**Output:** human activity categoryPreprocess the input data through the Butterworth low-pass filter and dividing by sensitivity coefficient k.Segment the data into overlapping windows.Extract the features and construct the feature vector x with normalization.Input x into three MGD models and calculate $p_{1}\left(x\right),\;p_{2}\left(x\right)$, and $p_{3}\left(x\right)$.if $p_{1}\left(x\right)==\underset{i=1,2,3}{\max}p_{i}\left(x\right)$: Output (LIA)else if $p_{2}\left(x\right)==\underset{i=1,2,3}{\max}p_{i}\left(x\right)$: Output (MIA)else if $p_{3}\left(x\right)==\underset{i=1,2,3}{\max}p_{3}\left(x\right)$ & $p_{3}\left(x\right)>\varepsilon_{2}$ & $TA<\varepsilon_{3}$: Output (VIA)else:Output (Fall)

## 4. Experimental Section

In this section, we validate our aforementioned methods. We start by introducing our experiment protocol, and then we specify the method for the experimental approach evaluation. 

We compare the recognition performance in terms of *F*1-measure, which represents the combination of precision and recall, for all the experiments. They are respectively defined as follows:

(13) $$Precision=\frac{T_{p}}{T_{p}+F_{p}}$$

(14) $$Recall=\frac{T_{p}}{T_{p}+F_{n}}$$

(15) $$F-\mathrm{measure}=\frac{2\times Recall\times Precision}{Recall+Precision}$$

where *T_n_* (true negatives) and *T_p_* (true positives) are the correct classifications of negative and positive examples, respectively. *F_n_* (false negatives) denotes the positive examples incorrectly classified into the negative classes. Inversely, *F_p_* (false positives) represents the negative examples incorrectly classified into the positive classes. Our proposed algorithm can classify human activities into four categories: LIA, MIA, VIA, and fall. F-measure is computed for each activity type. For example, the output of the algorithm can be treated as non-fall or fall when evaluating the capability of recognizing falls. In our study, the simulations were executed in a MATLAB 2017 environment, which was run on an ordinary PC with 2.60 GHz CPU and 4 Gb memory space.

### 4.1. Experiment Protocol

Table 1 offers the summary of different activities and activity categories and reports examples of activities that fall under each specific activity category. The definition principle of LIA, MIA, and VIA categories is shown in the table [45]. The Metabolic Equivalent of Task (MET) [46], a physiological measure gauging the energy cost of physical activities, can be applied to measure physical activities. For example, 1 MET is treated as the Resting Metabolic Rate (RMR) obtained during quiet sitting. MET values of activities range from 0.9 (sleeping) to 23 (running at 22.5 km/h). Specifically, MET values of light intensity activities are in the range of (1, 3), moderate intensity activities are in the range of (3, 6), and vigorous intensity activities are in the range of (6, 10). Assuming that a MET value is greater than 9, the user should be engaging intensely vigorous activity [21]. Additionally, we also list fall as an activity category in this table, divided into four types (forward falling, backward falls, left-lateral falling, right-lateral falling).

The conduction of the experiments lasted two weeks with our campus chosen as its location; 10 people (five males and five females) were randomly selected as participants. They were all informed of the purpose and procedure of the study. Their ages were in the range of (20, 45) years, and none of the participants presented an unhealthy status or limb injury. Their BMI measurements were in the range of (16, 34), and the BMI distribution in our experiment sample can be seen in Table 2.

During the experiment, the participants could choose the time period for each session freely. MPU6050 was used to collect the tri-axial acceleration and the tri-axial angular velocity with sampling rate of 50 Hz. As the waist is the geometric center of the human body [38], the sensor was attached to each person’s waist. The *x*-axis, *y*-axis, and *z*-axis of the sensor corresponded to the horizontal (transverse rotation), median (axial rotation), and lateral (sagittal rotation) movements of the human body, respectively. Participants were asked to perform the following activities: (1) working at a computer at a desk; (2) reading a book; (3) having a conversation (in calm status); (4) walking; (5) walking downstairs; (6) walking upstairs; (7) running; (8) rope jumping; (9) forward falling; (10) backward falling; (11) left-lateral falling; and (12) right-lateral falling. On average, experiment sessions (activities 1–8) lasted about 30 min and each participant had to perform 10 sessions over the two weeks. Each participant was asked to simulate each type of genuine fall 60 times on a safety cushion in order to validate the fall detection performance of the MGD-based classifier. In reality, each activity category contains more than the above examples. In our study, we used only one activity type in each category (LIA, MIA, and VIA) to train the MGDs, and used other activities to verify that the classifier can accurately classify the unknown activity. The ratio of the dataset for training and testing was 4:1. The datasets of activity 1, activity 4, and activity 7 were selected to train $p_{1}\left(x\right),\;p_{2}\left(x\right)$, and $p_{3}\left(x\right)$. In practice, users were asked to collect their own dataset for model adaption. The selected activities are the commonest activities among their belonging classes, which are more convenient for users to collect the data. The testing dataset included all of the activities in Table 1.

### 4.2. Classification Performance of Our Proposed Method

In this subsection, we firstly validate the performance of our method in recognizing LIA, MIA, VIA, and fall. Secondly, in order to evaluate the effect of adaption, the generic model is also trained. To train the personalized classifier, all steps of our algorithm are conducted. Both the training dataset and the testing dataset include only activity data from the selected subject. The data from the rest of the subjects are used to initialize the centroids in the automatic annotation step. The personalized classifier is also trained for all volunteers. On the part of the generic classifier, leave-one-out (LOO) cross-validation is utilized in training and testing the classifier. Thus, one participant’s dataset is left out and the parameters in the MGD models are defined based on the data from the others’ datasets. The trained classifier is then tested on the participant’s dataset that was left out earlier. The same procedure is repeated for the rest of participants. It is worth mentioning that the collected samples of fall were only included in the test dataset, because the proposed MGD classifier does not require fall data in its training phase. The performance evaluation for our proposed algorithm presented in Table 3 shows that 100% of the cells contain values higher than 0.95 and 38% contain values higher than 0.98. The result shows the best performance to be for LIA, with an F-measure of 0.9875 averaged for all participants. For MIA, VIA, and fall, the mean values of the F-measure reached 0.9729, 0.9692, and 0.9766, respectively.

**Figure 2:**
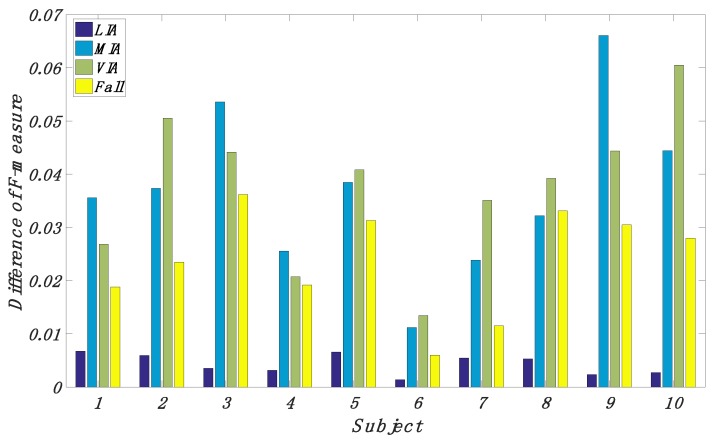
Difference in F-measure between a personalized classifier and a generic classifier for four recognized activity categories.

In Figure 2, the effect of personalization is shown. The difference is in favor of the personalized classifier for all participants. For nine of the participants, the difference is statistically significant. As shown in Figure 1, the improvement of performance for MIA, VIA, and fall is much more than that for LIA. The estimated averages of the improvement in F-measure are 0.0043, 0.0368, 0.0376, and 0.0238 for the four categories of activity recognition. The one exception’s BMI value is between 18.5 and 25. Moreover, we notice that the subjects whose BMI value is larger than 25 or less than 18.5 always have larger difference and we want to test whether any difference of proportions that we observe is significant. $u_{i}\left(i=1,\ldots 10\right)$ is set to be the average value of the improvement in F-measure for the ith subject. $u_{mean}$ is the average value of $u_{i}$ over all subjects. If the subjects’ BMI values are between 18.5 and 25, these subjects belong to the Normal group and the rest of them belong to the Abnormal group. If the subjects’ $u_{i}$ values are greater than $u_{mean}$, they are termed significant subjects (SS). Otherwise, they are termed insignificant subjects (IS). Then we assume the null hypothesis that the Normal group and the Abnormal group are equally likely to be SS. The distribution of SS and IS among the Normal group and the Abnormal group is summarized in Table 4. Through Fisher’s extract test [47], we can get *p*-value < 0.05, which evidently indicates that Normal group and Abnormal group are not equally to be SS.

**Figure 3:**
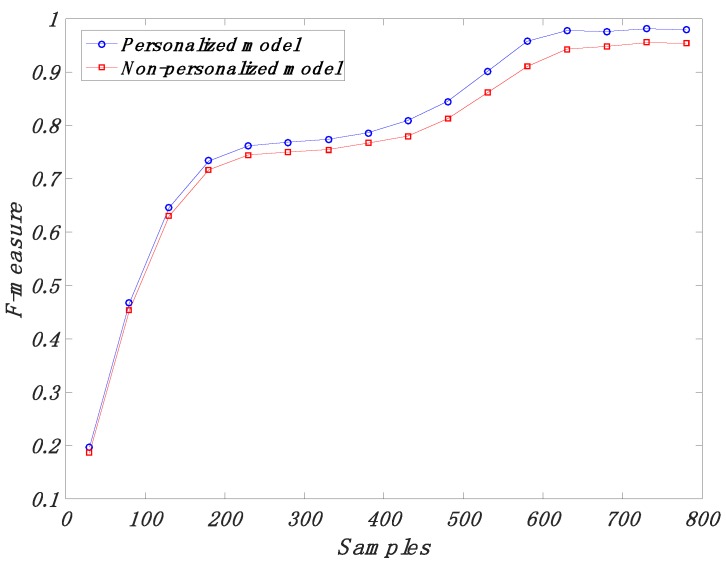
Comparison between personalized models and non-personalized models for different training dataset sizes.

In Figure 3, we compare the personalized model and the non-personalized model for different training dataset sizes. The curves show average F-measure values for each defined personal training dataset size and non-personal training dataset size. The average F-measure values for the personalized classifier converged at a higher average F-measure value of around 0.9796, while the non-personalized classifier only reached an F-measure value of around 0.9540. 

### 4.3. Comparison of the Proposed Algorithm with Previous Studies

In this subsection, we compare our method with the personalization algorithms proposed in the [29,31] as well as fall detection methods reported in [22,27]. The methods proposed in [29,31] have similar workflows for building a personalized classifier. Firstly, they train a generic model before transferring to a new user. Secondly, the trained model is used to classify the unlabeled data from the new user. Thirdly, confident samples are selected to reconstruct the previous model. The algorithms and parameter settings for both methods are summarized in Table 5. The simulations for SVM were conducted using LIBSVM [48] packages and J48 is simulated using WEKA toolbox [49]. K-Medoids, TrainsRKELM, and HMM were implemented by us using Matlab. The Gaussian kernel $K\left(x,x_{i}\right)=\exp\left(-x-x_{i}{}^{2}/\sigma \right)$ was used in SVM and RKELM in their studies. For the Gaussian kernel parameter σ, the generalized accuracy is estimated by using different parameter values $\sigma =\left[2^{-5},\ldots ,2^{20}\right]$, and choosing the value with the best performance. For RKELM, the generalized performance is estimated, using various combinations of regularized parameter λ and the subset size $\tilde{n}$: $\lambda =\left[2^{-5},\ldots ,2^{30}\right]$, and $\tilde{n}$ is increased gradually with an interval of 5. For SVM, the generalized performance was estimated using various combinations of parameters λ and K (the number of confident samples for each cluster): $\lambda =\left[2^{-5},\ldots ,2^{30}\right]$, and K is increased by an interval of 5. In order to compare with the HMM-based fall detection algorithm proposed in Reference [22], the HMM was initialized as follows: the number of invisible states M=3, the number of observation values N=8, and initial state distribution $\pi_{1}=1,\;\pi_{i}=0\left(i=2,\ldots ,M\right)$. Then, the generated performance was estimated using different thresholds of $P_{2}=\left[0.1,\ldots 0.2\right]$. For J48, the generated performance is estimated by using different combination of the pruning confidence C and the minimum number of instances per leaf M: C=[0.01,…,1] and M is increased by an interval of 1 Twenty trials of simulations for each aforementioned parameter or parameter combination were carried out. The best performances obtained are shown in this paper.

As can be seen from Table 6, our proposed method outperforms others in recognizing LIA, MIA, and VIA. The performance of fall detection is not the best among them, but its F-measure, 0.9766, is still competitive. Moreover, the fall samples are not required in our training phase. The training time in the table represents the time of adapting a model to a specific user’s dataset. In addition, the average training time (20 trials) of our method is 3.56 s, while the other two methods consume 10.12 s and 0.93 s. Moreover, the average testing time (20 trials) of our method is 0.02 s, while the other two methods consume 2.83 s and 0.15 s. This indicates that our method is much faster than the methods presented in [29,31] if embedded in real-time activity recognition applications. 

In Table 7, we compare the fall detection performance of our method with two efficient algorithms used in [22,27]. Our algorithm shows the highest F-measure values of 0.9766. The J48 algorithm [27] has the least training time of 2.03 s, but its F-measure value of 0.9410 is not competitive. Our proposed algorithm is the most efficient in testing phase with testing time of 0.02 s, while the others consume 0.04 s and 0.05 s. 

## 5. Conclusions

In conclusion, a user-adaptive algorithm for activity recognition based on K-Means clustering, LOF, and MGD is presented. In order to personalize our classifier, we proposed a novel initialization method for a K-Means algorithm that is based on a pre-collected dataset which has been already labeled. In our study, we used a pre-collected activity dataset to generate initial centroids of K-Means clustering. After the iterations, the dataset was clustered and labeled by the centroids. Then, LLOF was used to select high confidence samples for model personalization. 

Due to inadequate fall data used for training a supervised learning algorithm, anomaly detection can be employed for falls detection. MGD is an effective classification technique, requiring only a few computational steps either in training phases or activity classification processes. Thus, it has low computational complexity compared with some computation-demanding classifiers. Moreover, it can produce an accurate classification performance with carefully selected features. Three MGD models were trained by a specific user’s dataset. LIA, MIA, and VIA could be differentiated through comparing probability values, which were outputs of $p_{1}\left(x\right),\;p_{2}\left(x\right)$, and $p_{3}\left(x\right)$. $\varepsilon_{2}$ and $\varepsilon_{3}$ were set to distinguish falls from VIA.

The experimental results showed that our personalized algorithm can effectively detect LIA, MIA, VIA, and falls with F-measures of 0.9875, 0.9729, 0.9692, and 0.9766, respectively. Moreover, compared with the algorithms in the literature [22,27,29,31], our proposed algorithm has lower computational complexity in the testing phase, with a testing time of 0.02 s. 

In our future work, we plan to apply multi-sensor fusion techniques [50,51] and deep learning methods to detect more complicated abnormal behaviors, which can be put solid ground for the design and implementation of a real system for real-time physical activity recognition. Additionally, we will engage in testing our proposed method using genuine fall datasets. 

## Figures and Tables

**Table 1:** Categorization of different activities.

Activity Category	Description	Activities
Light intensity activity (LIA)	Users perform common daily life activities in light movement condition.	Working at a desk, reading a book, having a conversation
Moderate intensity activity (MIA)	Users perform common daily life activities in moderate movement condition.	Walking, walking downstairs, walking upstairs
vigorous intensity activity (VIA)	Users perform vigorous activities to keep fit.	Running, rope jumping
Fall	Users accidentally falls to the ground in a short time.	Forward falling, backward falling, left-lateral falling, right-lateral falling

**Table 2:** BMI distribution of the participants.

Description	Underweight	Normal	Overweight and Obese
BMI	<18.5	(18.5, 25)	≥25
Number of subjects	3	5	2

**Table 3:** F-measure of four recognized activity categories for our proposed algorithm. The last row presents the average values for all participants.

Users	LIA	MIA	VIA	Fall
**1**	0.9822	0.9766	0.9765	0.9709
**2**	0.9985	0.9821	0.9673	0.9635
**3**	0.9963	0.9782	0.9775	0.9836
**4**	0.9822	0.9673	0.9661	0.9750
**5**	0.9866	0.9687	0.9642	0.9809
**6**	0.9782	0.9821	0.9739	0.9774
**7**	0.9865	0.9680	0.9641	0.9887
**8**	0.9733	0.9753	0.9661	0.9850
**9**	0.9978	0.9653	0.9523	0.9711
**10**	0.9931	0.9651	0.9839	0.9723
**Mean**	**0.9875**	**0.9729**	**0.9692**	**0.9766**

**Table 4:** Distribution of SS and IS among Normal group and Abnormal group.

	Normal	Abnormal	Row total
SS	1	5	6
IS	4	0	4
Column total	5	5	10

**Table 5:** Details of two models proposed in literature.

Author	Algorithm	Parameter Setting
Viet et al. [29]	K-Medoids, SVM	$K=95,\lambda =2^{15},\sigma =2^{10}$
Deng et al. [31]	TransRKELM	$\sigma =2^{10},\tilde{n}=400,\lambda =2^{25}$
Tong et al. [22]	HMM	$M=3,N=8,\;\pi_{1}=1,\;\pi_{i}=0\left(i=2,\ldots ,M\right),\;P_{2}=0.123$
Shi et al. [27]	J48	C=0.25,M=2

**Table 6:** Performance of the user-adaptive methods.

Author	LIA	MIA	VIA	Fall	Training Time (s)	Testing Time (s)
Viet et al. [29]	0.9844	0.9230	0.9376	0.9845	10.12	2.83
Deng et al. [31]	0.9854	0.9423	0.9364	0.9813	0.93	0.15
Our proposed method	0.9875	0.9729	0.9692	0.9766	3.56	0.02

**Table 7:** Performance of the fall detection methods.

Author	Fall	Training Time (s)	Testing Time (s)
Tong et al. [22]	0.9508	4.02	0.04
Shi et al. [27]	0.9410	2.03	0.05
Our proposed method	0.9766	3.56	0.02
